# Bladder Wall Telangiectasia in a Patient with Ataxia-Telangiectasia and How to Manage?

**DOI:** 10.1155/2015/615368

**Published:** 2015-11-29

**Authors:** Fatma Deniz Aygün, Serdar Nepesov, Haluk Çokuğraş, Yıldız Camcıoğlu

**Affiliations:** Department of Pediatric Infectious Diseases, Clinical Immunology and Allergy, Istanbul University, Cerrahpasa Medical Faculty, 34098 Istanbul, Turkey

## Abstract

Ataxia-telangiectasia (A-T) is a rare neurodegenerative, inherited disease causing severe morbidity. Oculocutaneous telangiectasias are almost constant findings among the affected cases as telangiectasia is considered the main clinical finding for diagnosis. Vascular abnormalities in organs have been reported infrequently but bladder wall telangiectasias are extremely rare. We aimed to report recurrent hemorrhage from bladder wall telangiectasia in a 9-year-old boy with A-T who had received intravenous cyclophosphamide for non-Hodgkin's lymphoma. Since A-T patients are known to be more susceptible to chemical agents, we suggested that possibly cyclophosphamide was the drug which induced bladder wall injury in this patient.

## 1. Introduction

Ataxia-telangiectasia is an immunodeficiency syndrome with a wide spectrum of findings including progressive cerebellar ataxia, oculocutaneous telangiectasia, ionizing radiation hypersensitivity, susceptibility to developing lymphoreticular malignancy, and defects in DNA repair [1]. It is an autosomal recessive inherited disorder caused by mutations in the ATM gene, a serine/threonine kinase located on the long arm of chromosome 11, that activates proteins in cellular response to DNA damage [2]. Approximately 10% of A-T homozygotes develop malignancy, mostly of the lymphoid system, including Hodgkin's lymphoma [1, 3]. Telangiectasia is pathognomonic component of the disease, partially giving the disease its name. Oculocutaneous area is the most commonly affected, vascular involvement in other parts of the body have been reported [4], but bladder wall telangiectasia is extremely rare and reported only in a few patients. This report describes development of bladder wall telangiectasia and hemorrhagic cystitis in a patient with ataxia-telangiectasia who had been treated with cyclophosphamide for NHL.

## 2. Case Report

A 9-year–old boy was admitted to the Immunology Department due to recurrent pulmonary infections and meningitis. He was the son of nonconsanguineous parents with no known diseases. His developmental stages were delayed. His height and weight measurements were less than 3rd percentile. He had ataxia and ocular telangiectasia, without cutaneous involvement. He did not have any other cerebellar sign. Physical examinations of chest, abdomen, and cardiovascular system were in normal limits. Laboratory analysis including complete blood count and liver and renal function tests were all in normal ranges. Serum immunoglobulin G level was low, 427 mg/dL (normal range 780–1600), IgA was low, 32 mg/dL (normal range 70–400), but IgM was very high, 1330 mg/dL (normal range 40–320). Flow cytometric analysis of T, B, and NK cells revealed normal values. Serum alpha fetoprotein (AFP) was elevated to 196,45 IU/mL (normal range < 5.8 IU/mL) supporting the diagnosis of ataxia-telangiectasia, and cranial MRI revealed cerebellar atrophy. A-T was also subsequently confirmed by identification of c.7788G>A(p.Glu2596Glu) homozygous mutations in both alleles of the ATM gene.

At the age of 9, he developed B-cell NHL in the lower pole of left kidney. He was treated with R-CHOP protocol, including cyclophosphamide (CPA), doxorubicin, vincristine, and prednisolone, which achieved cure. Concomitant Mesna administration and massive hydration were given to prevent bladder damage during the course of treatment.

Six months after the cure, he developed massive, painless hematuria requiring blood transfusion for two times. His blood pressure was normal. In laboratory studies, blood urea nitrogen, creatinine level, and coagulation parameters were all normal; thrombocytopenia was not seen at any time. The polymerase chain reaction (PCR) analysis of urine for adenovirus and polyoma BK virus tests were both negative; urine culture was sterile. The viral serology including cytomegalovirus (CMV), Epstein-Barr virus, and CMV DNA was negative. No calculi or signs of damage at renal parenchyma were seen in ultrasonography. Cystoscopy revealed a blood clot in the bladder with multiple telangiectasias in the bladder wall. A large clot was removed from the bladder following catheter irrigation of the bladder with regular saline solution. Later on, bilateral ureteral stents were placed. We were able to perform PCR assay for polyomavirus JC in his serum during his follow-up and viremia was not demonstrated. During the 6th month of follow-up, he still had telangiectasia in bladder wall but a gradual improvement was observed in the patient's clinical symptoms with the ureteral stents and he did not need further urological treatment.

## 3. Discussion

Telangiectasia is the hallmark of ataxia-telangiectasia. In most cases, telangiectasias first appear when the child reaches three to five years of age; they are progressive and have symmetric distribution. In our patient, telangiectasia first appeared when he was 4 years old. He was diagnosed to have bladder wall telangiectasias when he was 9 years old.

Telangiectasias of blood vessels are seen primarily on the bulbar conjunctivae and on exposed areas of the skin, typically the pinnae, nose, face, and neck [5]. Vascular anomalies in various organs, like brain, hepatic vein, and intestinal mucosa, have been reported, but bladder wall telangiectasia is very rare.

Significant bleeding from telangiectasia is also very infrequent in AT. There are only few reported cases describing mucosal hemorrhage from cerebral telangiectasia besides the reported infrequent bladder wall hemorrhages before our case [6].

Bladder wall telangiectasia was first described in 2008 with two patients of A-T and lymphoma who also received CPA including chemotherapy [7]. Authors suggested that hematuria could be due to the toxic effect of CPA during lymphoma therapy. They concluded that since A-T is a DNA repair disorder and the ATM gene has an important role in cell cycle arrest in response to cellular stress, it is possible that A-T patients are more sensitive than normal individuals to CPA induced bladder damage which leads to the development of clinically significant telangiectasias.

In the same year, another patient with A-T who developed massive hematuria due to bladder wall varices was reported from Japan [8]. He was also treated with CPA for autoimmune thrombocytopenia 3 years ago.

Patients with A-T and diagnosis of malignancy are more susceptible to toxic effects of chemotherapy. Despite significant toxicity, CPA remains as a drug of choice in cancer chemotherapy. The exact mechanism causing the induction of cell death is unknown, but Goldstein et al. considered that DNA interstrand cross-links can be responsible for its cytotoxicity. They also showed the apoptotic death induced by mafosfamide, a CPA analogue by contribution of DNA replication and transcription inhibition [9].

Hemorrhagic cystitis is a well-known side effect of CPA caused by exposure of bladder mucosa to acrolein, a urinary toxic metabolite of CPA. The damage of bladder wall induced by acrolein is followed by ulceration, neovascularization, and hemorrhage which leads to hemorrhagic cystitis. As A-T patients are more sensitive to chemical agents, acrolein may accelerate the development of telangiectasia in bladder wall mucosa.

Sandoval and Swift reported hemorrhagic cystitis in 50% of A-T patients receiving 1200 mg/m^2^ or more CPA despite hyperhydration [10], but these patients did not receive Mesna which is known to reduce bladder toxicity of CPA. Concomitant administration of Mesna with CPA is recommended as we did.

It is suggested that some of the patients with A-T who receive CPA therapy may subsequently develop bladder hemorrhage regardless of the single or cumulative dose. So the use of CPA, even at a relative low dose, should be avoided in the treatment of patients with A-T.

The last case of bleeding from bladder wall telangiectasia in A-T patient was reported by Christmann et al. in 2008. The patient was a 10-year-old boy having bladder wall telangiectasia and infection with polyomavirus JC. BK and JC virus are from the Polyomaviridae family and cause hemorrhagic cystitis in immunocompromised patients. But the patient reported in that article had also received CPA for lymphoma like other cases [11]. We investigated our patient for polyomavirus, adenovirus, and cytomegalovirus and therefore excluded these viral causes.

In conclusion, A-T is caused by mutations in the ATM gene, which is involved in the detection of DNA damage, and plays an important role in cell cycle progression. The defect in DNA repair in A-T also explains the sensitivity of patients to chemotherapeutic agents. It is possible that our case developed hemorrhagic cystitis after CPA therapy. Despite our support of massive hydration and Mesna administration, his underlying defective DNA damage was the probable cause of bleeding.
